# Cachexia in preclinical rheumatoid arthritis: Longitudinal observational study of thigh magnetic resonance imaging from osteoarthritis initiative cohort

**DOI:** 10.1002/jcsm.13533

**Published:** 2024-06-24

**Authors:** Kamyar Moradi, Bahram Mohajer, Ali Guermazi, C. Kent Kwoh, Clifton O. Bingham, Soheil Mohammadi, Xu Cao, Mei Wan, Frank W. Roemer, Shadpour Demehri

**Affiliations:** ^1^ Department of Musculoskeletal Radiology, Russell H. Morgan Department of Radiology and Radiological Science Johns Hopkins University School of Medicine Baltimore MD USA; ^2^ Department of Radiology Boston University School of Medicine Boston MA USA; ^3^ University of Arizona Arthritis Center University of Arizona College of Medicine Tucson AZ USA; ^4^ Department of Medicine, Division of Rheumatology Johns Hopkins University Baltimore MD USA; ^5^ School of Medicine Tehran University of Medical Sciences Tehran Iran; ^6^ Department of Orthopedic Surgery Johns Hopkins University School of Medicine Baltimore MD USA; ^7^ Department of Radiology Universitätsklinikum Erlangen & Friedrich‐Alexander‐Universität Erlangen‐Nürnberg Erlangen Germany

**Keywords:** Adiposity, Magnetic resonance imaging (MRI), Muscle composition, Preclinical rheumatoid cachexia

## Abstract

**Background:**

Preclinical rheumatoid arthritis (Pre‐RA) is defined as the early stage before the development of clinical RA. While cachexia is a well‐known and potentially modifiable complication of RA, it is not known if such an association exists also in the Pre‐RA stage. To investigate such issue, we aimed to compare the longitudinal alterations in the muscle composition and adiposity of participants with Pre‐RA with the matched controls.

**Methods:**

In this observational cohort study, the Osteoarthritis Initiative (OAI) participants were categorized into Pre‐RA and propensity score (PS)‐matched control groups. Pre‐RA was retrospectively defined as the absence of RA from baseline to year‐2, with progression to physician‐diagnosed clinical RA between years 3–8 of the follow‐up period. Using a validated deep learning algorithm, we measured MRI biomarkers of thigh muscles and adiposity at baseline and year‐2 follow‐ups of the cohort. The outcomes were the differences between Pre‐RA and control groups in the 2‐year rate of change for thigh muscle composition [cross‐sectional area (CSA) and intramuscular adipose tissue (Intra‐MAT)] and adiposity [intermuscular adipose tissue (Inter‐MAT) and subcutaneous adipose tissue (SAT)]. Linear mixed‐effect regression models were used for comparison.

**Results:**

After 1:3 PS‐matching of the groups for confounding variables (demographics, risk factors, co‐morbidities, and knee osteoarthritis status), 408 thighs (102 Pre‐RA and 306 control) of 322 participants were included (age mean ± SD: 61.7 ± 8.9 years; female/male: 1.8). Over a 2‐year period, Pre‐RA was associated with a larger decrease in total thigh muscle CSA [estimate, 95% confidence interval (CI): −180.13 mm^2^/2‐year, −252.80 to −107.47, *P*‐value < 0.001]. Further examination of thigh muscle composition showed that the association of the presence of Pre‐RA with a larger decrease in muscle CSA over 2 years was noticeable in the quadriceps, flexors, and sartorius muscle groups (*P*‐values < 0.05). Comparison of changes in total adipose tissue showed no difference between Pre‐RA and control participants (estimate, 95% CI: 48.48 mm^2^/2‐year, −213.51 to 310.47, *P*‐value = 0.691). However, in the detailed analysis of thigh adiposity, Pre‐RA presence was associated with a larger increase in Inter‐MAT (estimate, 95% CI: 150.55 mm^2^/2‐year, 95.58 to 205.51, *P*‐value < 0.001).

**Conclusions:**

Preclinical rheumatoid arthritis is associated with a decrease in muscle cross‐sectional area and an increase in intermuscular adipose tissue, similar to rheumatoid cachexia in clinical rheumatoid arthritis. These findings suggest the presence of cachexia in the preclinical phase of rheumatoid arthritis. Given that cachexia, which can exacerbate health outcomes, is potentially modifiable, this study emphasizes the importance of early identification of patients in their preclinical phase.

## Introduction

Rheumatoid arthritis (RA) is a systemic autoimmune and inflammatory disease that primarily causes damage to the cartilage and bones within joints.^1^ Beside affecting the joints, up to one‐third of RA patients also suffer from skeletal muscle mass and function loss while retaining their adipose tissue mass, a condition known as rheumatoid cachexia.^2^ Rheumatoid cachexia is associated with worsening muscle strength and functional capacity and can contribute to long‐term morbidity and decreased life expectancy.^3^, ^4^ A recent meta‐analysis revealed that disease‐modifying anti‐rheumatic drugs (DMARDs) had no significant effect on skeletal muscle mass in patients already diagnosed with RA.^5^ However, rheumatoid cachexia may be modifiable with interventions, such as specific diets^6^, ^7^, ^8^ and intensive physical exercises^9^ [S1, S2], especially if detected at the early stages. Current diagnostic criteria for rheumatoid cachexia primarily rely on measurements of fat‐free and fat masses [S3, S4]. This contrasts with classic cachexia, which is marked by significant weight loss, especially in muscle mass, and increased protein breakdown due to an underlying condition such as severe and uncontrolled RA. The criteria for classic cachexia typically include a weight loss of ≥5% within a year (or a BMI ≤ 20 kg/m^2^) and at least three of the following: diminished muscle strength, fatigue, decreased appetite, low fat mass index, and abnormal biochemistry (elevated inflammatory markers, anaemia, and low serum albumin) [S5]. Consistent with these criteria, the estimated prevalence of rheumatoid cachexia in RA patients ranges from 13.3% to 30.0%, yet none of these patients meet the criteria for classic cachexia.^10^

Early identification and treatment of individuals at risk (i.e., preclinical) or in the initial stages of RA can significantly aid in preventing disease progression.^11^ To detect those with early‐stage RA, various clinical and ancillary assessments have been suggested^1^, ^12^ [S6, S7]. In RA, inflammation onset is a gradual process involving various stages, including genetic predisposition, exposure to environmental factors, systemic autoimmunity related to RA, symptoms like joint pain without evident clinical arthritis signs, undifferentiated arthritis, and eventual RA development.^13^ The 2010 European League Against Rheumatism Standing Committee on Investigative Rheumatology (EULAR) recommended terminology refers to all these stages as preclinical RA (Pre‐RA), a term used retrospectively for those who have some RA‐related symptoms and will eventually develop clinical RA.^14^ Besides, a taskforce from EULAR has defined a set of clinical characteristics for patients with arthralgia who are at risk of progressing to clinical RA. These characteristics include, but are not limited to, morning stiffness, joint symptoms of recent onset (duration <1 year), most severe symptoms present in the early morning, and the presence of a first‐degree relative with RA,^15^ providing a strong foundation for screening the patients with inflammatory symptoms at their preclinical stage.

The excessive production of inflammatory cytokines and systemic autoimmunity has been identified as major contributors to the development of rheumatoid cachexia.^16^, ^17^ Meanwhile, it has been shown that systemic inflammation and autoimmunity are also present at the preclinical stage of the disease and are involved in the pathogenesis of RA^18^, ^19^ [S8, S9]. This suggests that the process of cachexia may begin silently and prior to the development of clinical RA. Although there are a few cross‐sectional studies suggesting presence of cachexia in early clinical RA (<6–12 months of duration), as measured by dual energy X‐ray absorptiometry (DXA),^20^, ^21^ the evidence regarding its manifestation in the preclinical stage of the disease remains notably scarce. This lack of evidence raises questions about the potential presence of cachexia in the preclinical stage preceding clinical RA onset. If cachexia does indeed manifest during the preclinical stage, it could signify a critical opportunity to integrate imaging assessments of skeletal muscle composition into the screening protocols for Pre‐RA individuals. Such an approach could pave the way for the early identification of at‐risk individuals and the implementation of targeted secondary preventive interventions aimed at mitigating the progression of cachexia and associated complications in RA.

Magnetic resonance imaging (MRI) is recognized as a valuable tool for detecting skeletal muscle loss associated with RA.^22^ The available thigh MRI images in Osteoarthritis Initiative (OAI) cohort are a useful resource and may be used as a surrogate to evaluate whole‐body skeletal muscle loss.^23^ This study aimed to investigate the association between Pre‐RA and cachexia by evaluating longitudinal changes in MRI‐derived biomarkers of muscle mass and adiposity in participants with Pre‐RA. The study's findings carry significant implications for researchers, clinicians, and patients. For researchers, it offers insights into the early stages of RA‐related cachexia. Clinicians can use this knowledge to enhance screening protocols and implement timely interventions, potentially improving patient outcomes. Patients stand to benefit from early detection and tailored preventive measures, mitigating long‐term complications of cachexia in RA. Ultimately, this study underscores the importance of targeted interventions in halting the progression of cachexia and improving care for RA patients.

## Methods

### Study population and design

In this longitudinal observational cohort study, data were obtained from the OAI cohort database, which is a multicentre longitudinal cohort of 4796 subjects (both knees in each subject) with or at risk of knee OA (2004–2015 ClinicalTrials.gov identifier: NCT00080171). The participants were recruited from five clinical centres (The Ohio State University; University of Maryland; Johns Hopkins University; University of Pittsburgh; Brown University/Memorial Hospital of Rhode Island). The study was Health Insurance Portability and Accountability Act‐compliant (HIPAA)‐compliant and has received ethics board approval by the institutional review board at the University of California, San Francisco (OAI Coordinating Center; approval number: 10‐00532), and all enrolled participants provided written informed consent [S10]. Names and details of the OAI datasets used for this study are available in Appendix S1. The OAI study included men and women aged 45 to 79 from all ethnic groups. However, those with physician‐diagnosed inflammatory arthritis, advanced knee OA, unable to have an MRI, or unwilling to participate were excluded from the cohort at baseline.

### Preclinical rheumatoid arthritis assessments: Exposure variable

**Figure 1:**
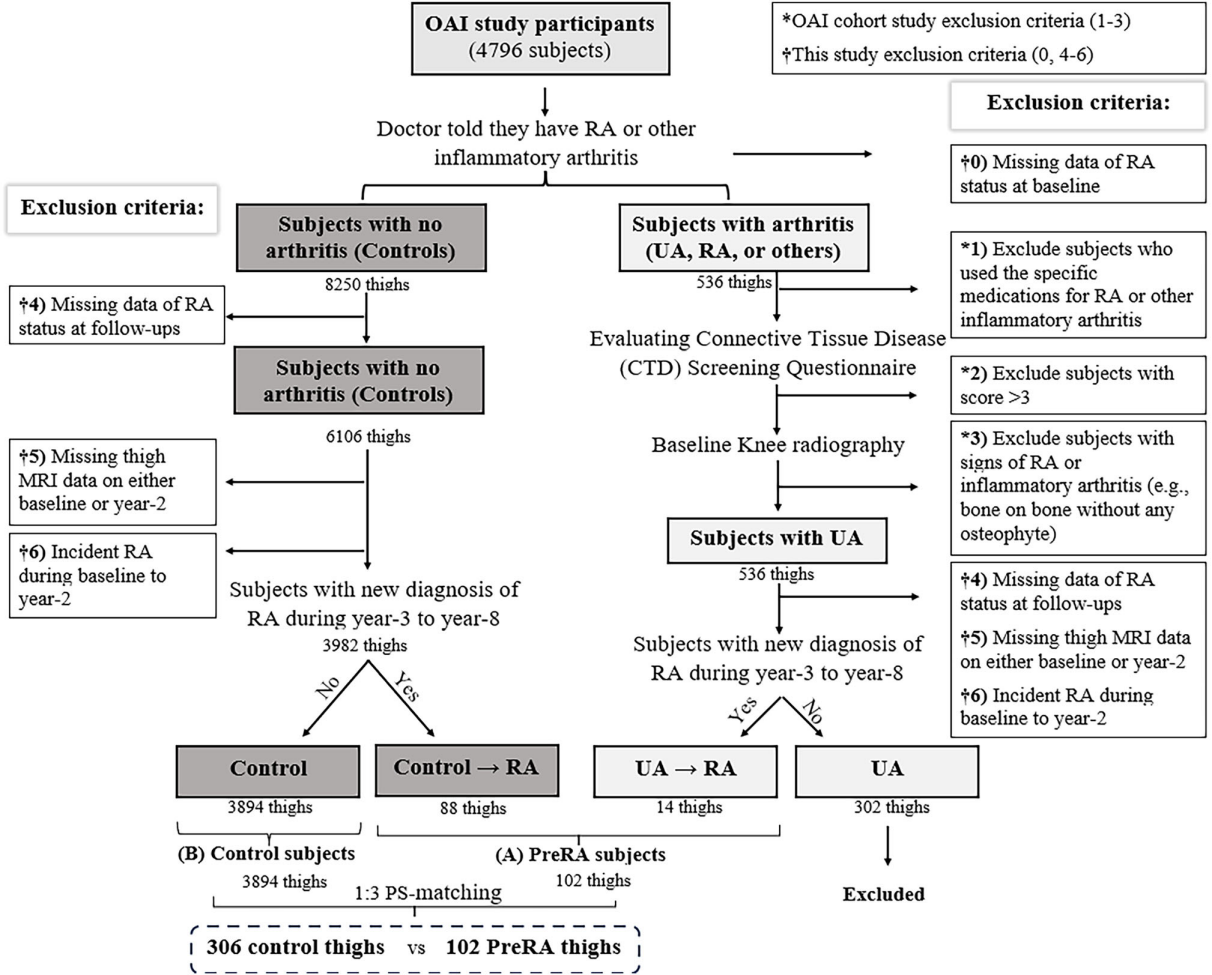
Flowchart showing the stepwise selection process. The exclusion criteria were applied in two stages: (1) participants with baseline RA/inflammatory arthritis were excluded during the screening visit of the OAI cohort (exclusions #1–3), and (2) additional exclusions (exclusions 0, 4–6) were implemented at the enrolment visit for this study.

**Figure 2:**
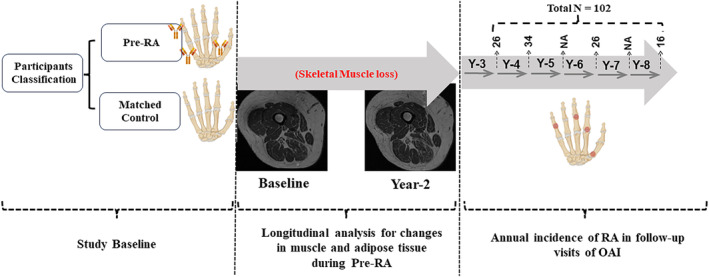
Diagram showing timing of the study assessments. Pre‐RA was retrospectively defined as OAI patients who progressed to RA during the 8‐year cohort follow‐up. Number of participants with progression to RA is shown at each year follow up. Pre‐RA, preclinical rheumatoid arthritis.

For this study, we selected participants who were diagnosed with preclinical rheumatoid arthritis (Pre‐RA) and control participants who were matched based on their propensity scores (PS). Pre‐RA was retrospectively defined as participants who had no RA diagnosis at baseline and did not develop RA during the 2‐year study period, but later progressed to clinical RA, according to physician diagnosis, during the follow‐up period from year‐3 to year‐8. We excluded participants who developed RA during the study period (baseline to year‐2 visits) as our goal was to investigate changes in body composition at the Pre‐RA stage, not in clinical RA. The OAI participants were included based on OAI protocol. For this study, which is derived from the OAI database, two expert rheumatologists (C. O. B. and C. K. K.) and three expert radiologists (A. G., F. W. R., and S. D.) made a secondary analysis of the publicly available database that was accumulated across the four clinical centres of OAI. To select these participants and their matched controls, we used the following inclusion/exclusion criteria (Figure 1):

1. At the baseline screening visit, participants were evaluated for the presence RA or other types of inflammatory arthritis. They were asked if a doctor had ever diagnosed them with RA or other inflammatory arthritis. Subjects who answered ‘no’ were considered to have no history of inflammatory arthritis and were classified as normal subjects. On the other hand, subjects who answered ‘yes’ were further evaluated with additional questions.
2. Subjects who answered ‘yes’ to the question about being diagnosed with RA or other inflammatory arthritis were asked about their use of specific medications, including methotrexate, plaquenil, enbrel, remicade, arava, gold, prednisone, sulfasalazine, anakinra, adalimumab, and infliximab. If they had used any of these treatments, they were excluded from the OAI cohort (exclusion 1: RA positive at baseline screening visit).
3. Subjects who reported being diagnosed with arthritis but had not used any of the mentioned medications were assessed for RA‐related symptoms using the connective tissue disease (CTD) screening questionnaire from the Nurses' Health Study (Appendix S2).^24^ This questionnaire has excellent specificity and sensitivity in detecting RA (Appendix S3). Subjects with a total score of 4 or more on the CTD questionnaire at baseline were considered to have RA and were excluded from the OAI cohort (exclusion 2: RA positive at baseline screening visit).
4. In addition, participants were considered to have inflammatory arthritis and were excluded from the OAI cohort if they showed signs of inflammatory arthritis in their baseline radiographs (severe joint space narrowing score or bone‐on‐bone without any osteophyte in that knee) (exclusion 3: RA positive at baseline screening visit).
5. After excluding all possible subjects with RA or inflammatory arthritis at baseline, the remaining participants that had the history of physician‐diagnosed arthritis were classified as having ‘undifferentiated arthritis (UA)’.
6. Participants with missing or poor‐quality MRI data at either the baseline or the two‐year timepoint, or those with missing data on RA follow‐up status, were further excluded. (exclusions 4 and 5: missing data).
7. According to the definition of the Pre‐RA term, any control or UA + subjects at baseline enrollment visit who developed RA during year‐3 to year‐8 follow‐up visits were categorized as belonging to the Pre‐RA group (Figure 2). Those who developed RA between the baseline and year‐2 were excluded (exclusion 6).

### Propensity‐score matching

To reduce bias from missing data [S11], we used the Little's test to evaluate the pattern of missing data and estimated missing values using multiple imputation (Appendix S4). Upon the completion of data imputation, a PS model with a 1:3 ratio was constructed for each dataset. The association between Pre‐RA and the longitudinal changes in outcome measures was subsequently investigated within each PS‐matched dataset. Finally, the results were aggregated across all datasets, adhering to the principles outlined in Rubin's rules, using the ‘within’ matching method [S12]. Several potential confounders were adjusted in the analysis, such as demographics (age and gender), risk factors (physical activity, BMI, body surface area, smoking, alcohol use, abdominal obesity, and dyslipidaemia), co‐morbidities (diabetes mellitus, malignancy, COPD, kidney failure, heart failure, and liver failure), and indices of KOA structural and symptomatic status (KL grade, JSN grade, and WOMAC total score) (Appendix S5). The accuracy of the matching between groups was assessed using the standardized mean difference (SMD), with a value of ≥0.1 indicating an imbalance.

### Quantitative MRI biomarkers of thigh muscle and adipose tissue: Primary outcome variables

According to the prospectively designed OAI protocol, axial T1‐weighted thigh MR images were acquired at baseline and year‐2 (Figure 2), using four 3T MRI scanners across the four clinical centers (Trio, Siemens Healthcare). Components of the protocol were optimized for the segmentation of subcutaneous and intermuscular fat depots, skeletal muscle, and specific muscle groups. Owing to the stringent guidelines applied to the scanners, the consistency of images across these centres and over time within each centre was ensured. Detailed protocol and standardization procedures can be found in https://nda.nih.gov/oai/study‐details.

To reflect 3D thigh muscle volume and composition, the axial slice representing the 33% distal length of the femur bone was selected for segmentation, taking into account variations in body height and thigh length.^25^, ^26^ A fully automated deep learning algorithm with a 2D U‐Net structure was developed and validated for segmentation and interpretation of thigh MRIs obtained from the OAI.^27^ The Intra‐muscular adipose tissue (Intra‐MAT) and inter‐muscular adipose tissue (Inter‐MAT) were calculated by summing white pixels inside and between muscle segments, respectively. The cross‐sectional area (CSA) for thigh muscles and subcutaneous adipose tissue (SAT) was calculated directly from segmentations and multiplied by pixel area to calculate CSAs (Appendix S7). Total thigh adipose tissue CSA was calculated by summing SAT, Intra‐MAT, and Inter‐MAT.

### Anthropometric assessments: Secondary outcome variables

To evaluate the association of Pre‐RA with indicators of general and central adiposity, we compared the longitudinal changes in BMI (baseline, year‐1, year‐2) and waist‐to‐height ratio (baseline, year‐2) between the groups, respectively.

### Data analysis

Statistical analysis was performed using the R software version 4.0.3 (packages: *haven*, *MatchIt*, *mitools, mice*, *lme4*, and *lmerTest*). Repeated measures analysis with multilevel linear mixed‐effect regression models were used to compare the rate of change in biomarkers of thigh muscle and adipose tissues over 2 years between participants with Pre‐RA and PS‐matched control participants and results were reported as rate of change difference, 95% confidence interval (CI). Random intercept was considered for each cluster of PS‐matched thighs, as well as for within‐subject similarities (due to the inclusion of thighs/knees of both sides). We primarily conducted the analysis based on thighs data (rather than participants) to account for the localized periarticular effects of the ipsilateral knee joint OA status on thigh muscles. Interaction of the time and presence of Pre‐RA (Pre‐RA × Time) was considered as the independent predictor, while thigh MRI biomarkers and anthropometric assessments were the dependent outcomes. The *P*‐values were adjusted for multiple comparisons using the Benjamini & Hochberg procedure. A two‐tailed adjusted *P*‐value of <0.05 was considered significant. We calculated the reliable change index (RCI), which provides a cut‐off for clinical significance.^28^, ^29^ The RCI is calculated as $\frac{X1-X2}{\sqrt{2}\times \mathrm{SEM}}$. X2 and X1 were the outcome values at year‐2 and baseline and SEM was the standard error of measurement (i.e., standard deviation divided by square root of the sample size). Calculated RCIs lower than −1.96 and higher than 1.96 [95% confidence interval (CI)] were considered as the reliable change (i.e., change that is larger than that due to measurement error alone).

## Results

### Characteristics of study participants

Initially, all 4796 participants of the OAI were included in the study, as individuals with established RA were excluded from this cohort. Out of the 6814 thighs with available good‐quality thigh MRIs at baseline and follow‐ups and also with available data of RA status and knee KL grade at baseline, 536 had a previous history of arthritis and were considered as thighs of participants with UA, while the remaining 8250 were considered controls. After excluding thighs of participants with missing data of RA status at follow‐ups, missing MRI data at either baseline or year‐2, and those with incident RA during the first 2 years of the study, we were left with 316 thighs of participants with UA and 3982 control thighs (Figure 1). We further excluded thighs of participants with UA but no progression to RA (UA‐non‐RA) and included 102 thighs of participants with Pre‐RA (88 control → RA, 14 UA → RA) and 3894 control thighs of participants with no further progression to RA. The final population for this study was achieved after 1:3 PS‐matching to account for potential confounders (Pre‐RA: Control 102: 306). The matching results showed no significant difference between the two groups (all SMDs <0.1), as shown in Table 1.

**Table 1:** Baseline characteristics of the study sample before and after propensity score matching according presence of pre‐RA

Variables	All OAI study participants' thighs (first imputed dataset)		SMD	PS‐matched participants' thighs (1: 3 ratio) (first matched dataset)		SMD
	Pre‐RA (−)	Pre‐RA (+)		Pre‐RA (−)	Pre‐RA (+)	
	*N* = 3894	*N* = 102		*N* = 306	*N* = 102	
Subject characteristics						
Age (year) [mean (SD)]	60.55 (8.80)	61.71 (9.03)	**0.129**	61.89 (9.39)	61.71 (9.03)	0.020
Sex (female %)	2206 (56.7)	66 (64.7)	**0.165**	198 (64.7)	66 (64.7)	0.000
Risk factors						
PASE score [mean (SD)]	166.51 (79.54)	163.84 (77.47)	0.034	169.21 (84.25)	163.84 (77.47)	0.066
BMI (kg/m^2^) [mean (SD)]	28.08 (4.56)	28.46 (4.90)	0.080	28.45 (4.82)	28.46 (4.90)	0.002
BSA (m^2^) [mean (SD)]	0.19 (0.04)	0.18 (0.04)	**0.121**	0.19 (0.04)	0.18 (0.04)	0.041
Abdominal (central) obesity [*N* (%)]^a^	3352 (86.1)	92 (90.2)	**0.128**	274 (89.5)	92 (90.2)	0.022
Dyslipidaemia [*N* (%)]	926 (23.8)	30 (29.4)	**0.128**	81 (26.5)	30 (29.4)	0.066
Alcohol use (≥1/week) [*N* (%)]	3240 (83.2)	76 (74.5)	**0.214**	225 (73.5)	76 (74.5)	0.022
Smoking status [*N* (%)]	837 (21.5)	14 (13.7)	**0.205**	41 (13.4)	14 (13.7)	0.010
Co‐morbidities						
Diabetes [*N* (%)]	211 (5.4)	15 (14.7)	**0.312**	50 (16.3)	15 (14.7)	0.045
COPD [*N* (%)]	78 (2.0)	4 (3.9)	**0.113**	12 (3.9)	4 (3.9)	0.000
Malignancy [*N* (%)]	143 (3.7)	0 (0.0)	**0.276**	0 (0.0)	0 (0.0)	0.000
Heart failure [*N* (%)]	50 (1.3)	6 (5.9)	**0.249**	22 (7.2)	6 (5.9)	0.053
Liver failure [*N* (%)]	3 (0.1)	2 (2.0)	**0.188**	3 (1.0)	2 (2.0)	0.082
Kidney failure [*N* (%)]	40 (1.0)	2 (2.0)	0.077	8 (2.6)	2 (2.0)	0.044
Knee joint osteoarthritis status						
WOMAC total score [mean (SD)]	9.05 (12.76)	19.25 (19.92)	**0.610**	18.49 (19.32)	19.25 (19.92)	0.039
KL grade [*N* (%)]			**0.269**			0.090
Grade 0	1591 (40.9)	31 (30.4)		91 (29.7)	31 (30.4)	
Grade 1	738 (19.0)	18 (17.6)		52 (17.0)	18 (17.6)	
Grade 2	995 (25.6)	30 (29.4)		90 (29.4)	30 (29.4)	
Grade 3	459 (11.8)	18 (17.6)		62 (20.3)	18 (17.6)	
Grade 4	111 (2.9)	5 (4.9)		11 (3.6)	5 (4.9)	
JSN grade [*N* (%)]			**0.184**			0.099
Grade 0	2626 (67.4)	62 (60.8)		196 (64.1)	62 (60.8)	
Grade 1	824 (21.2)	25 (24.5)		63 (20.6)	25 (24.5)	
Grade 2	369 (9.5)	14 (13.7)		45 (14.7)	14 (13.7)	
Grade 3	75 (1.9)	1 (1.0)		2 (0.7)	1 (1.0)	
RA‐related symptoms^b^						
Positive total CTD score [*N* (%)]^c^	0 (0.0)	8 (7.8)	**0.413**	0 (0.0)	8 (7.8)	**0.413**
Physician‐diagnosed arthritis [*N* (%)]^c^	0 (0.0)	14 (13.7)	**0.561**	0 (0.0)	14 (13.7)	**0.561**
NSAIDs use for joint pain or arthritis more than half the days of the past month^b^						
Prescription NSAIDs (%)	197 (5.0)	8 (7.8)	**0.114**	14 (4.6)	8 (7.8)	**0.135**
Non‐prescription NSAIDs (%)	654 (16.8)	26 (25.5)	**0.214**	53 (17.3)	26 (25.5)	**0.200**

Data are presented in numbers of thighs. A significant difference for SMD was defined as ≥0.1 and is shown in bold.

BMI, body mass index; BSA, body surface area; CTD, connective tissue disease; JSN, joint space narrowing; KL, Kellgren and Lawrence; NSAIDs, nonsteroidal anti‐inflammatory drugs; PASE, Physical Activity for Elderly Scale; Pre‐RA, preclinical rheumatoid arthritis; PS, propensity score; SMD, standardized mean difference; SD, standard deviation; WOMAC, Western Ontario and McMaster Universities Osteoarthritis.

^a^ Abdominal obesity was defined as a waist circumference of ≥94 cm in men and ≥80 cm in women on physical examination according to international diabetes foundation criteria.

^b^ Not included in the PS‐matching.

^c^ Positive total CTD score was defined as score ≥1 in the CTD screening questionnaire. Physician‐diagnosed arthritis was defined according to the question ‘Has doctor ever said you have RA or other inflammatory arthritis’ and was revised according to the study inclusion/exclusion criteria to exclude those with clinical RA at baseline.

The final sample had an average age of 61.7 ± 8.9 years (mean ± SD) and a gender ratio of 1.8 (female/male). Finally, participants with Pre‐RA exhibited higher rates of RA‐related symptoms and a greater use of NSAIDs for arthralgia or arthritis at baseline, compared to the control participants (Table 1).

### Comparison of the longitudinal changes in MRI biomarkers of thigh muscles

**Figure 3:**
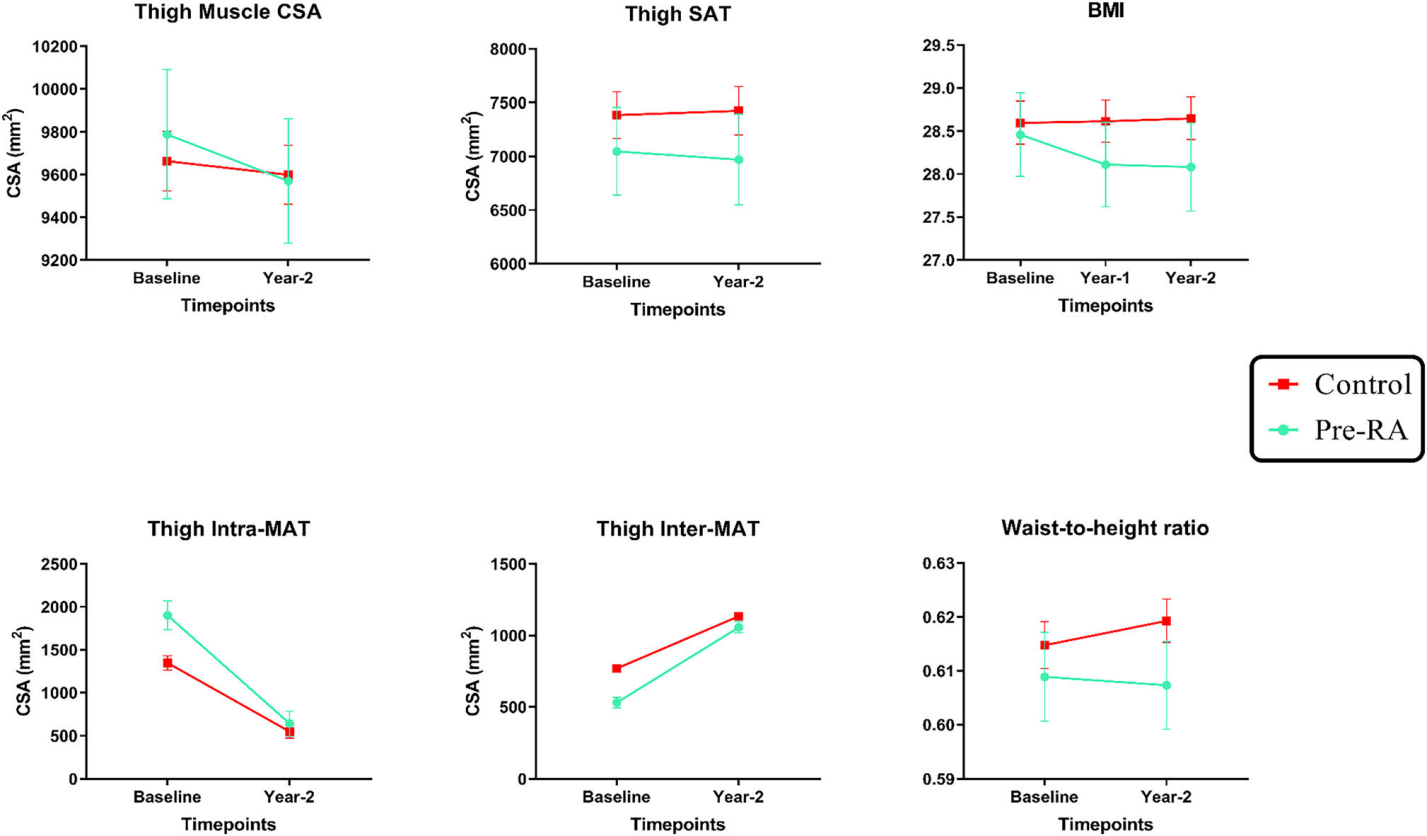
Comparison of 2‐year changes in biomarkers of muscle and adipose tissue between participants with baseline diagnosis of pre‐RA and PS‐matched control participants. Data are presented in mean ± standard error mean (SEM). Mixed‐effect regression models of changes in MRI‐derived biomarkers showed that the presence of pre‐RA (green line and symbol) is associated with a larger decrease in the total thigh muscle CSA and a larger increase in inter‐MAT deposition, compared to the control (red line and symbol). Furthermore, the presence of pre‐RA was found to be associated with changes in BMI and waist‐to‐height ratio levels over 2 years. BMI, body mass index; CSA, cross‐sectional area; Inter‐MAT, inter‐muscular adipose tissue; Intra‐MAT, intra‐muscular adipose tissue; Pre‐RA, preclinical rheumatoid arthritis; PS, propensity score; SAT, subcutaneous adipose tissue.

Over a period of 2 years, as shown in Figure 3, the presence of Pre‐RA at baseline was associated with a larger decrease in muscle CSA (estimate, 95% CI, RCI: −180.13 mm^2^/2‐year, −252.80 to −107.47, −3.73), but no significant difference in Intra‐MAT deposition (estimate, 95% CI, RCI: 28.64 mm^2^/2‐year, −199.60 to 256.88, 0.19). Table 2 presents the results of a comparison of MRI biomarkers for each thigh muscle group. In summary, the association between Pre‐RA and decreased muscle CSA was present in quadriceps, flexors, and sartorius. Furthermore, the 2‐year changes in Intra‐MAT of all muscle groups were statistically similar between Pre‐RA and control groups, except for the sartorius (estimate, 95% CI, RCI: −26.66 mm^2^/2‐year, −41.59 to −11.73, −2.70).

**Table 2:** Comparison of longitudinal 2‐year changes in mass biomarkers of muscle and adipose tissues between thighs of participants with preclinical RA and thighs of PS‐matched controls

Outcome variables	% Of baseline value^b^	Rate of change difference per 2‐year (95% CI)^a^	*P* value	RCI
Anthropometric biomarkers				
BMI (kg/m^2^)	−1.75%	−0.50 (−0.72, −0.28)	<0.001	**−3.17**
Waist‐to‐height ratio (%)	−1.37%	−0.84 (−1.41, −0.27)	0.004	**−2.03**
Quadriceps				
Muscle CSA (mm^2^)	−2.07%	−101.51 (−141.01, −62.01)	<0.001	**−3.87**
Intra‐MAT CSA (mm^2^)	−0.42%	−2.50 (−109.75, 104.77)	0.960	−0.03
Adductors				
Muscle CSA (mm^2^)	−2.65%	−30.70 (−57.22, −4.18)	0.013	−1.76
Intra‐MAT CSA (mm^2^)	16.75%	38.08 (−5.79, 81.95)	0.060	1.33
Flexors				
Muscle CSA (mm^2^)	−1.05%	−33.69 (−57.88, −9.50)	0.003	**−2.09**
Intra‐MAT CSA (mm^2^)	3.73%	19.71 (−58.45, 97.86)	0.595	0.37
Sartorius				
Muscle CSA (mm^2^)	−3.92%	−14.23 (−19.02, −9.46)	<0.001	**−4.43**
Intra‐MAT CSA (mm^2^)	−23.26%	−26.66 (−41.59, −11.73)	0.001	**−2.70**
Total thigh				
Muscle CSA (mm^2^)	−1.87%	−180.13 (−252.80, −107.47)	<0.001	**−3.73**
Intra‐MAT CSA (mm^2^)	1.95%	28.64 (−199.60, 256.88)	0.790	0.19
Inter‐MAT CSA (mm^2^)	22.14%	150.55 (95.58, 205.51)	<0.001	**4.21**
SAT CSA (mm^2^)	−1.79%	−130.70 (−250.39, −11.01)	0.032	−1.51
Total adipose tissue CSA (mm^2^)	0.51%	48.48 (−213.51, 310.47)	0.691	0.28

CI, confidence interval; CSA, cross‐sectional area; Inter‐MAT, inter‐muscular adipose tissue; Intra‐MAT, intra‐muscular adipose tissue; PS, propensity score; RA, rheumatoid arthritis; RCI, reliable change index; SAT, subcutaneous adipose tissue.

^a^ Longitudinal mixed‐effect regressions were used to assess the difference in muscle biomarkers between the PS‐matched groups. Random intercept was considered for clusters of matched participants and clusters of thighs for each participant.

^b^ To represent the effect size, the ratio of change rate difference to baseline value was calculated for each biomarker.

### Comparison of the longitudinal changes in the detailed MRI biomarkers of adiposity

A detailed analysis of thigh adiposity showed that Pre‐RA is associated with a larger increase in Inter‐MAT (estimate, 95% CI, RCI: 150.55 mm^2^/2‐year, 95.58 to 205.51, 4.21) (Table 2, Figure 2). Although Pre‐RA was associated with a larger decrease in SAT deposition (estimate, 95% CI: −130.70 mm^2^/2‐year, −250.39 to −11.01), this finding was not reliable (RCI = −1.51). When comparing the total adipose tissue, there was no difference in the change of total adipose tissue CSA over 2 years between the groups (estimate, 95% CI, RCI: 48.48 mm^2^/2‐year, −213.51 to 310.47, 0.28).

### Comparison of the longitudinal changes in anthropometric biomarkers of adiposity

As presented in Table 2 and Figure 3, mixed‐effect regression models showed that presence of Pre‐RA is associated with a larger 2‐year decrease in BMI (estimate, 95% CI, RCI: −0.50 kg/m^2^/2‐year, −0.72 to −0.28, −3.17) and waist‐to‐height ratio (estimate, 95% CI, RCI: −0.84%/2‐year, −1.41 to −0.27, −2.03).

## Discussion

Our study demonstrated that Pre‐RA presence correlates with longitudinal muscle CSA decline prior to the diagnosis of clinical RA. Our MRI analysis revealed no change in total adipose tissue, along with a larger increase in Inter‐MAT, associated with Pre‐RA. The differences observed between the Pre‐RA and control groups may not be attributed to the baseline characteristics of the participants, as rigorous PS‐matching was performed to adjust for potential confounders.

Rheumatoid cachexia is characterized by the loss of skeletal muscle mass despite normal BMI and excess adiposity. Skeletal muscle loss in rheumatoid cachexia is thought to occur secondary to chronic systemic inflammation and reduced physical activity due to joint pain and stiffness. The observation of similar muscle loss in the preclinical stage of RA could be due to the presence of inflammatory processes before the clinical onset of the disease, as well as the presence of some symptoms in a proportion of participants. In terms of the adiposity component, associated increase in the Inter‐MAT deposition might potentially be attributed to muscle atrophy, which results in the replacement of tissue with adipose tissue. Further research is needed to understand the exact mechanisms behind these findings. The increased deposition of Inter‐MAT may worsen the outcome of patients with Pre‐RA and RA as is associated with development of chronic systemic inflammation, insulin resistance, increased total cholesterol and decreased mobility and function.^30^

Screening Pre‐RA patients for cachexia poses clinical and socioeconomic challenges. To measure the changes in body composition and estimate the extent of cachexia, researchers have used a variety of techniques such as bioelectrical impedance analysis (BIA), DXA, and potassium‐40 whole body counting.^31^ However, there are several advantages for CT/MRI examinations over these techniques. First, due to limited availability and high costs of whole‐body imaging techniques, there has been a shift toward limited CT and MRI scans. For instance, CT scan of the lumbar vertebrae or MRI of the mid‐thigh are known as a consistent reference point for analysing body composition, including skeletal muscle mass and adipose tissue.^23^, ^32^, ^33^ Second, while widespread assessment of body composition using techniques like DXA has been useful in detecting the loss of skeletal muscle mass and compensation by gain in whole‐body adipose tissue, cross‐sectional imaging techniques such as CT and MRI provide a more accurate approximation of skeletal muscles and adipose tissue anatomy, allowing for differentiation between visceral and subcutaneous adipose tissues.^34^ To our knowledge, this is the first report on adipose tissue detail in the RA spectrum, a finding made possible by the advantages provided by MRI. Third, a significant advantage of cross‐sectional imaging techniques is that they are commonly used in various research and clinical settings and can be easily measured in large numbers through manual and automated (deep learning‐based) segmentation methods [S13‐S15], and therefore, may be a practical candidate for screening subjects with Pre‐RA for their muscle status. For instance, we utilized the thigh MRIs of the OAI cohort, which is primarily designed for studying OA, as a database also rich in clinical information about RA, developed a deep learning algorithm to measure longitudinal changes in MRI biomarkers of skeletal muscle and adipose tissue,^34^ and employed the generated database of biomarkers to subjects with Pre‐RA. However, it should also be noted that the applicability of our approach in a clinical environment may be limited by the high standardization of the OAI images and the complexity of the deep learning algorithm that we used to extract the information. Furthermore, establishing the normal values and cut‐off points for muscle variations between healthy individuals and those with Pre‐RA is crucial in order to accurately diagnose cachexia at this early stage.

Progress toward curing and preventing RA has shifted focus to the ‘window of opportunity’ in early stages, before patients exhibit characteristics that would classify them as having clinical RA. In this study, participants with Pre‐RA represented higher rates of RA‐related symptoms (according to the CTD screening questionnaire) and a greater use of NSAIDs for arthralgia or arthritis at baseline compared to control participants. Although some participants with Pre‐RA showed symptoms at baseline, it is important to note that CTD scores are only available for the baseline timepoint of the OAI study, making it impossible to evaluate development of these symptoms in other participants throughout the rest of the cohort until progression to RA. However, existing literature consistently supports the presence of inflammatory symptoms at the preclinical stage and their role as an indicator for the need for further screening steps, such as measuring serum biomarkers of inflammation and autoimmunity.^13^, ^15^ The American College of Rheumatology (ACR) has introduced a new International Classification of Diseases (ICD)‐10 code, R76.81, for Pre‐RA. This code delineates Pre‐RA as the presence of abnormal RA‐related immunologic findings without a current or prior diagnosis of clinically apparent inflammatory arthritis. Since cachexia was observed at the preclinical stage of the disease, it may be helpful to consider screening for body composition (i.e., skeletal muscle mass and adiposity) and monitoring changes in these measures during the preclinical stage of RA, similar to the approach used in clinical RA cases.

Cachexia is a multifactorial condition characterized by weight loss, muscle wasting, and increased protein breakdown due to underlying chronic illnesses such as malignancy (cancer cachexia), chronic heart failure (cardiac cachexia), chronic obstructive pulmonary disease (COPD), acquired immunodeficiency syndrome (AIDS), and autoimmune disorders like RA (rheumatoid cachexia). Notably, loss of skeletal muscle has been observed up to 18 months prior to the clinical diagnosis of pancreatic cancer.^35^ Pre‐cachexia, a probable early stage of the syndrome, is the focus of research, where (multi‐modal) interventions may slow down the process of cachexia, as there are limited treatments for the condition in advanced stages.^36^ While no research has been conducted on pre‐cachexia in the context of RA, it is possible that rheumatoid cachexia could be similarly modified, particularly if detected at an early stage. Treatment with either synthetic or biological DMARDs has not been successful in managing rheumatoid cachexia.^5^ This may be likely because these treatments are usually started too late to prevent the condition from developing, and since they are not anabolic, they are unable to restore normal body composition.^37^ However, previous research suggests that rheumatoid cachexia can be modified if the right intervention is chosen and applied at the appropriate time. For instance, Marcora et al. showed that both etanercept and methotrexate were equally effective in stabilizing early rheumatoid cachexia.^38^ Similarly, Book et al. discovered that the initiation of DMARDs in patients with early RA was associated with a reduction in the change of lean body mass over a 2‐year period.^39^ Additionally, of the available adjunct treatments, high‐intensity exercises have shown the greatest benefit in increasing skeletal muscle mass and decreasing adiposity^9^ [S1, S2]. Due to the low participation of patients with RA in regular and high‐intensity exercises, because of the disease symptoms as well as misconceptions about the safety and benefits of physical activity, clinicians may become interested in exploring additional treatment strategies, such as the use of specific dietary supplements, to modify body composition.^6^, ^7^, ^8^

This study has several limitations. First, although we observed the presence of cachexia in patients with Pre‐RA, translating this finding to the clinic presents several challenges. As there is no screening tool available (e.g., elevated serum biomarkers of systemic inflammation and autoimmunity) in the OAI for detecting participants with Pre‐RA and CTD data for detecting early symptoms was limited to the baseline timepoint, we defined Pre‐RA retrospectively as participants who progressed to clinical RA in the future. Future studies with prospective design are required to define Pre‐RA based on presence of inflammatory symptoms not meeting clinical RA or serum biomarkers of autoimmunity. Second, since the 2010 criteria for Pre‐RA requires serum biomarkers of systemic inflammation, which are not provided in the OAI study, we had to use the CTD questionnaire as a tool highly correlated with the ACR 1987 revised criteria for classification of RA.^40^ In this regard, the definitions of arthritis in this study were limited to self‐reported questions in the OAI dataset, and laboratory tests would improve accuracy. Third, Since the OAI study did not include whole‐body imaging assessments such as CT, MRI, or DXA, we used longitudinal thigh MRIs (optimized for skeletal muscle and adipose tissue segmentation) as biomarkers to represent changes in overall muscle composition. The OAI study was chosen for its large sample size, long‐term follow‐up, and availability of robust demographic, clinical, and ancillary data. This allowed for rigorous PS‐matching to address potential confounding bias. While not a gold‐standard method for demonstrating generalized changes in muscle throughout the body, we adjusted for local periarticular effects of concomitant knee OA (i.e., KL grade, JSN grade, and WOMAC total score) to better demonstrate the association between Pre‐RA and ‘generalized’ longitudinal cachexia. Further studies using other datasets and body imaging methods are also needed to measure such association. Fourth, our segmentation algorithm is specifically trained for a certain anatomical location, namely the 33% distal level of the femur bone (distal‐proximal). It does not provide volumetric quantitative analysis of the thigh muscles due to the absence of isotropic volumetric voxels in the existing OAI MRI protocol.^27^ Nevertheless, prior research on the OAI dataset has depicted the 33% distal length of the femur bone, a key anatomical landmark in MRI, as this area's assessments are indicative of the 3D volume and composition of the thigh muscle.^25^, ^26^ Finally, the limited sample size, observational design, and susceptibility to selection bias of this study restrict the generalizability of its results. Further clinical trials are needed to address the narrative of this study and confirm its findings.

In conclusion, this study demonstrated that the presence of preclinical rheumatoid arthritis is associated with a larger decrease in muscle mass and a larger increase in intermuscular adipose tissue. These findings suggest the possible presence of cachexia during the preclinical stage of rheumatoid arthritis. Given that rheumatoid cachexia, which can exacerbate health outcomes, may be addressable through targeted dietary adjustments and exercise regimes, this study emphasizes the importance of early identification of patients in the preclinical stage of rheumatoid arthritis. Future directions for this study should focus on several key areas to advance understanding and clinical application. Further investigation is warranted to elucidate the causal relationship between preclinical rheumatoid arthritis and cachexia, enhance screening methods for preclinical rheumatoid arthritis and associated cachexia, identify systemic factors at play, and downstream clinical trials to implement early secondary interventions such as dietary adjustments and exercise regimes to mitigate cachexia prior to RA clinical diagnosis. Lastly, longitudinal studies with robust methodologies (e.g., volumetric thigh muscle assessment) are warranted to confirm and expand upon the findings of this study, addressing limitations such as sample size, observational design, and selection bias.

## Funding

This research was supported by the NIH National Institute on Aging (NIA) under award number P01AG066603 and NIH National Institute of Arthritis and Musculoskeletal and Skin Diseases (NIAMS) under award number R01AR079620‐01.

## Conflict of interest

AG is a shareholder of BICL and consultant to Pfizer, TissueGene, Pfizer, Novartis, Coval, ICM, TrialSpark, and Medipost. FWR is shareholder of BICL, LLC, and consultant to and Grünenthal GmbH. SD reported that he received funding from Toshiba Medical Systems (for consultation) and grants from GERRAF and Carestream Health (for a clinical trial study). The views expressed are those of the authors and not necessarily those of the National Health Service, the NIHR, or the Department of Health. None of the authors has any conflicting personal or financial relationships that could have influenced the results of this study. Other authors declare that they did not have any competing interests. The authors of this manuscript certify that they comply with the ethical guidelines for authorship and publishing in the *Journal of Cachexia, Sarcopenia and Muscle* [S16].

## Supporting information


**Appendix S1:** Names and details of the OAI datasets used for the study.
**Appendix S2:** Connective tissue disease (CTD) screening questionnaire.
**Appendix S3:** Comparison between CDT and 1987 RA Questionnaires (filled by all UA).
**Appendix S4:** The pattern of missing data Little's test.
**Appendix S5:** Variables included in the propensity‐score matching.
**Appendix S6:** Segmentation and quantification of MRI‐derived thigh biomarkers.

## Data Availability

The de‐identified clinical and demographic information of subjects is publicly available at the osteoarthritis initiative project data repository at https://oai.nih.gov. The R codes used in this work are available from the corresponding author upon reasonable requests.
