# Supplementary material for: Cachexia in preclinical rheumatoid arthritis: Longitudinal observational study of thigh magnetic resonance imaging from osteoarthritis initiative cohort
Source: J Cachexia Sarcopenia Muscle. 2024 Jun 24;15(5):1823–33. doi: 10.1002/jcsm.13533 (PMC11446725; doi:10.1002/jcsm.13533)
Supplement: Supplementary file 1 — Appendix S1: Names and details of the OAI datasets used for the study. Appendix S2: Connective tissue disease (CTD) screening questionnaire. Appendix S3: Comparison between CDT and 1987 RA Questionnaires (filled by all UA). Appendix S4: The pattern of missing data Little's test. Appendix S5: Variables included in the propensity‐score matching. Appendix S6: Segmentation and quantification of MRI‐derived thigh biomarkers. [file JCSM-15-1823-s001.docx]

# Cachexia in Preclinical Rheumatoid Arthritis: Longitudinal Observational Study of Thigh MRIs from Osteoarthritis Initiative CohortAppendix 1: Names and details of the OAI datasets used for the study

**Supplemental Table 1.** Osteoarthritis Initiative (OAI) datasets used in the analysis.

| **Dataset** | **Filename** | **Release version** |
| --- | --- | --- |
| All clinical | allclinical00 | 0.2.2 |
| (data regarding all clinical information) | allclinical01 | 1.2.1 |
|  | allclinical03 | 3.2.1 |
|  | allclinical05 | 5.2.1 |
|  | allclinical06 | 6.2.1 |
|  | allclinical08 | 8.2.1 |
|  | Allclinical10 | 10.2.1 |
| MRI tracking and QA | mri00 | 0.2.2 |
| (data regarding availability of MRI and | mri03 | 3.2.1 |
| quality assessment) | mri06 | 6.2.1 |
| Enrollees | Enrollees | 25 |
| (data regarding baseline enrollment of OAI participants) |  |  |
| Knee X-ray semi-quantitative reading (Kxr sq) | Kxr sq 00 | 0.8 |

KOA: Knee osteoarthritis, OAI: Osteoarthritis initiative, QA: quality assessment

# Appendix 2: Connective tissue disease (CTD) screening questionnaire

**Supplemental Table 2.** CTD Questionnaire

| No. | Question and/or variable definition | Yes/No | Score |
| --- | --- | --- | --- |
| Q1 | In the morning, have you ever had joint stiffness in any joints lasting at least one hour? | Yes/No | 1/0 |
| Q1a | Did you have this morning stiffness for more than 6 weeks? | Yes/No | 1/0 |
| Q2 | Have you ever had nodules or bumps under the skin around the elbow or ankle? | Yes/No* | - |
| Q3 | Have you ever had swelling in any of the following joints lasting more than 6 weeks? | Yes/No | - |
| Q3a (R) | Any finger or thumb of right hand | Yes/No | - |
| Q3a (L) | Any finger or thumb of left hand | Yes/No | - |
| Q3b (R) | Wrist or elbow of right hand | Yes/No | - |
| Q3b (L) | Wrist or elbow of left hand | Yes/No | - |
| Q3c (R) | Right knee | Yes/No | - |
| Q3c (L) | Left knee | Yes/No | - |
| Q4 | Are there 3 or more "Yes" responses to Questions 1a, 2, 3a, 3b, 3c | - | 1/0 |
| Q5 | Is there at least one "Yes" response for any wrist or finger in Question 3a and 3b? | - | 1/0 |
| Q6 | Are both RIGHT and LEFT marked "Yes" for any one of the above joint categories, in Questions 3a, 3b, 3c | - | 1/0 |
| Q7 | Have you ever had positive blood test for rheumatoid arthritis? | Yes/No | 1/0 |
| Total score # | |  | **Range:0-6** |

* The final score of this question has not been specifically mentioned in OAI study.

# Subjects with total score > 3 were considered as rheumatoid arthritis (RA) and were excluded in the OAI; subjects with score of 0-3 were considered as unclassified arthritis (UA).

# Appendix 3: Comparison between CDT and 1987 RA Questionnaires (filled by all UA)

|  | **CDT questionnaires score** | **1987 RA Questionnaires** | Correlation, P-value |
| --- | --- | --- | --- |
| **Score** |  |  |  |
| **0** | 300 | 296 | 0.952, P-value < 0.001 |
| **1** | 173 | 178 |  |
| **2** | 48 | 66 |  |
| **3** | 53 | 16 |  |

Correlation, Pearson

It should be noted that subjects who had a negative history of any sign and/or symptoms of arthritis did not fill the form and it has been assumed that these subjects had zero score for all CTD questionnaire variables. In brief, UA-non-RA and UA-RA have filled the form and answered above questions.

**Supplementary Table 3.** 1987 RA criteria Questionnaire

| **No.** | **Question and/or variable definition** | **Yes/No** | **Score** |
| --- | --- | --- | --- |
| **Q1** | **Morning stiffness:** lasting at least 1 hour at least for 6 weeks | Yes/No | 1/0 |
| **Q2** | **Arthritis of ≥ 3 joint areas:** At least 3 joint areas simultaneously have had swelling or fluid lasting more than 6 weeks. | Yes/No | 1/0 |
| **Q3** | **Arthritis of hand joints:** Have you ever had swelling in finger or thumb or wrist lasting more than 6 weeks? | Yes/No | 1/0 |
| **Q4** | **Symmetric arthritis:** Simultaneous involvement of the same joint areas on both sides of the body lasting more than 6 weeks. | Yes/No | 1/0 |
| **Q5** | **Rheumatoid nodules:** Have you ever had nodules or bumps under the skin around the elbow or ankle? | Yes/No* | 1/0 |
| **Q6** | **Serum rheumatoid factor:** Have you ever had positive blood test for rheumatoid arthritis? | Yes/No | 1/0 |
| **Q7** | **Radiographic changes:** Radiographic changes typical of rheumatoid arthritis | Yes/No# | 1/0 |
| **Total score #** | |  | **Range: 0-7** |

* The final score of this question has not been mentioned explicitly in OAI study.

# Subjects with a total ≥4 were considered as rheumatoid arthritis (RA); subjects with a score of 0-3 were considered as unclassified arthritis (UA).

# Appendix 4: The pattern of missing data Little’s test

We assessed the pattern of missing data using a test of missing completely at random (Little’s test), visual representation, and logistic regression models. The results showed a non-random pattern for missing data in the OAI dataset [S17], with fewer than 2.1% missing values for all matching variables (Supplemental Table 4). Despite the missing not at random data pattern, we included all matching variables in multiple imputation models, used according to previous studies to efficiently minimize the possible associated bias [S18, S19].

**Supplemental Table 4.** Percentage of missing data of the covariate included in the multiple imputations and PS-matching methods.

| **Variables** | **Missing %** |
| --- | --- |
| **Subject characteristics** |  |
| **Age** | 0.00% |
| **No. of women** | 0.00% |
| **Body surface area** | 1.61% |
| **Risk factors** |  |
| **PASE score** | 0.44% |
| **BMI** | 0.07% |
| **Abdominal (central) obesity** | 2.68% |
| **Dyslipidemia** | 0.00% |
| **Alcohol use** | 0.34% |
| **Smoking** | 0.40% |
| **Comorbidities** | |
| **Diabetes** | 1.44% |
| **Malignancy** | 1.03% |
| **COPD** | 1.73% |
| **Kidney dysfunction** | 1.90% |
| **Advanced liver dysfunction** | 0.83% |
| **Heart failure** | 0.73% |
| **Knee Osteoarthritis status** | |
| **WOMAC total score** | 0.38% |
| **JSN grade** | 0.72% |
| **KL grade** | 0.72% |

BMI: Body Mass Index, JSN: Joint Space Narrowing, KL: Kellgren-Lawrence grade, PASE: Physical Activity for Elderly Scale, PS: Propensity-score, WOMAC: Western Ontario and McMaster Universities Osteoarthritis.

# Appendix 5: Variables included in the propensity-score matching

PS-matching variables included age (in years), gender (female/male), body-mass index (BMI, Weight/(Height)^2^ in Kg/m^2^), physical activity scale for the elderly score (PASE), Body surface area (calculated using the Mosteller method [S20]: BSA (m^2^) = [weight (kg) × height (cm)/3600]^1/2^), abdominal obesity (defined as a waist circumference of ≥94 cm in men and ≥80 cm in women according to international diabetes foundation criteria [S21], yes/no), alcohol consumption (number of participants with ≥1/week use), smoking (current or past history of smoking, yes/no), diabetes (either self-reported diabetes or use of oral or injective diabetes medications, yes/no), dyslipidemia (using lipid-lowering medications as indicated in the participants’ MIF at baseline, yes/no), malignancy (self-reported history of cancer, other than skin cancer, leukemia or lymphoma, yes/no), chronic obstructive pulmonary disease (self-reported, having emphysema, chronic bronchitis, or chronic obstructive lung disease, yes/no), heart failure (self-reported history of having heart failure or receiving treatment for heart failure, yes/no), kidney dysfunction (self-reported, ever had problem with kidneys, poor kidney function based on blood high creatinine, yes/no), advanced liver dysfunction (self-reported, have cirrhosis or serious liver damage, yes/no), WOMAC total score, KL grade (based on baseline knee X-ray, grade 0/1/2/3/4), and whole JSN grade (based on baseline knee X-ray, grade 0/1/2/3). Units, levels, and categories of variables are also listed in Table 1 in the main text.

# Appendix 6: Segmentation and quantification of MRI-derived thigh biomarkers

**Supplemental Figure 1.**  Illustration of study markers of thigh MRI.

**
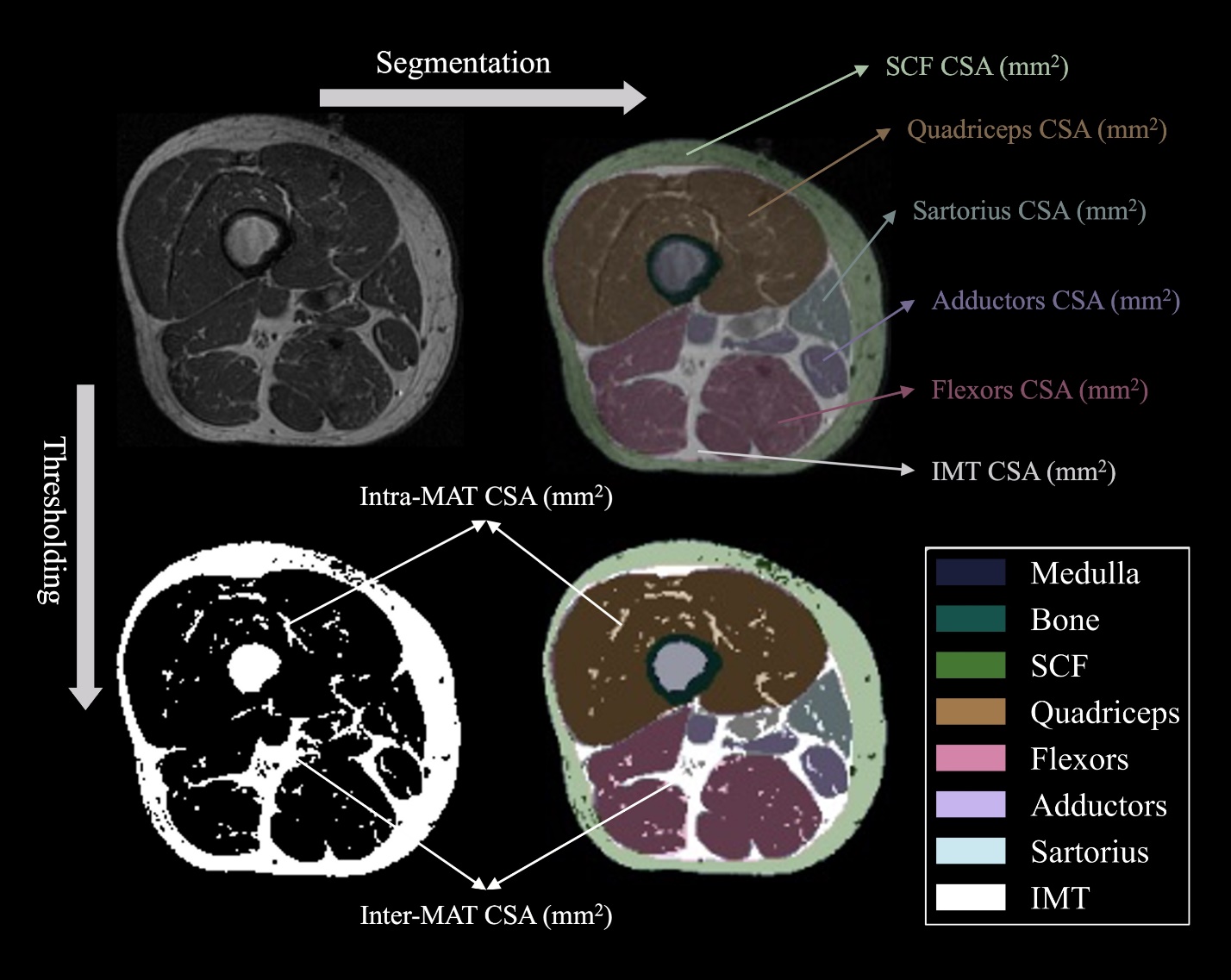
**

CSA: Cross-sectional Area, IMT: Inter-muscular Tissue, Inter-MAT: Inter-muscular Adipose Tissue, Intra-MAT: Intra-muscular Adipose Tissue, SCF: Subcutaneous Fat

# Supplemental References

S1. Marcora SM, Lemmey AB, Maddison PJ. Can progressive resistance training reverse cachexia in patients with rheumatoid arthritis? Results of a pilot study. J Rheumatol 2005;**32**:1031-1039.

S2. Metsios GS, Lemmey A. Exercise as Medicine in Rheumatoid Arthritis: Effects on Function, Body Composition, and Cardiovascular Disease Risk. Journal of Clinical Exercise Physiology 2015;**4**:14-22.

S3. Engvall IL, Elkan AC, Tengstrand B, Cederholm T, Brismar K, Hafström I. Cachexia in rheumatoid arthritis is associated with inflammatory activity, physical disability, and low bioavailable insulin‐like growth factor. Scandinavian journal of rheumatology. 2008;37(5):321-8.

S4. Elkan A-C, Håkansson N, Frostegård J, Cederholm T, Hafström I. Rheumatoid cachexia is associated with dyslipidemia and low levels of atheroprotective natural antibodies against phosphorylcholine but not with dietary fat in patients with rheumatoid arthritis: a cross-sectional study. Arthritis research & therapy. 2009;11:1-11.

S5. Evans WJ, Morley JE, Argilés J, Bales C, Baracos V, Guttridge D, et al. Cachexia: a new definition. Clinical nutrition. 2008;27(6):793-9.

S6. Arnett FC, Edworthy SM, Bloch DA, McShane DJ, Fries JF, Cooper NS, et al. The American Rheumatism Association 1987 revised criteria for the classification of rheumatoid arthritis. Arthritis Rheum 1988;**31**:315-324.

S7. Kay J, Upchurch KS. ACR/EULAR 2010 rheumatoid arthritis classification criteria. Rheumatology 2012;**51**:vi5-vi9.

S8. van Tuyl LH, Stack RJ, Sloots M, van de Stadt LA, Hoogland W, Maat B, et al. Impact of Symptoms on Daily Life in People at Risk of Rheumatoid Arthritis. Musculoskeletal Care 2016;**14**:169-173.

S9. Harre U, Georgess D, Bang H, Bozec A, Axmann R, Ossipova E, et al. Induction of osteoclastogenesis and bone loss by human autoantibodies against citrullinated vimentin. J Clin Invest 2012;**122**:1791-1802.

S10. Nevitt M, Felson D, Lester G. The osteoarthritis initiative. Protocol for the cohort study 2006;**1**:

S11. Donders AR, van der Heijden GJ, Stijnen T, Moons KG. Review: a gentle introduction to imputation of missing values. J Clin Epidemiol 2006;**59**:1087-1091.

S12. Rubin DB. Multiple imputation. In: Chapman and Hall/CRC; 2018. pp. 29-62.

S13. Pishgar F, Shabani M, Quinaglia ACST, Bluemke DA, Budoff M, Barr RG, et al. Adipose tissue biomarkers and type 2 diabetes incidence in normoglycemic participants in the MESArthritis Ancillary Study: A cohort study. PLoS Med 2021;**18**:e1003700.

S14. Pishgar F, Shabani M, Quinaglia ACST, Bluemke DA, Budoff M, Barr RG, et al. Quantitative Analysis of Adipose Depots by Using Chest CT and Associations with All-Cause Mortality in Chronic Obstructive Pulmonary Disease: Longitudinal Analysis from MESArthritis Ancillary Study. Radiology 2021;**299**:703-711.

S15. Hathaway Q, Ibad HA, Bluemke DA, Pishgar F, Kasaiean A, Klein JG, et al. Predictive Value of Deep Learning-derived CT Pectoralis Muscle and Adipose Measurements for Incident Heart Failure: Multi-Ethnic Study of Atherosclerosis. Radiology Cardiothoracic imaging 2023;**5**:e230146.

S16. von Haehling S, Morley JE, Coats AJS, Anker SD. Ethical guidelines for publishing in the journal of cachexia, sarcopenia and muscle: update 2017. Journal of cachexia, sarcopenia and muscle 2017;8:1081-1083.

S17. Roderick JAL. A Test of Missing Completely at Random for Multivariate Data with Missing Values. Journal of the American Statistical Association 1988;**83**:1198-1202.

S18. Resseguier N, Giorgi R, Paoletti X. Sensitivity Analysis When Data Are Missing Not-at-random. Epidemiology 2011;**22**:282.

S19. Schafer JL, Graham JW. Missing data: our view of the state of the art. Psychol Methods 2002;**7**:147-77.

S20. Mosteller RD. Simplified calculation of body-surface area. N Engl J Med 1987;**317**:1098.

S21. Alberti KG, Zimmet P, Shaw J. Metabolic syndrome--a new world-wide definition. A Consensus Statement from the International Diabetes Federation. Diabet Med 2006;**23**:469-480.
